# Strategies for the Enhancement of Secondary Metabolite Production via Biosynthesis Gene Cluster Regulation in *Aspergillus oryzae*

**DOI:** 10.3390/jof10050312

**Published:** 2024-04-25

**Authors:** Xiao Jia, Jiayi Song, Yijian Wu, Sai Feng, Zeao Sun, Yan Hu, Mengxue Yu, Rui Han, Bin Zeng

**Affiliations:** 1 College of Pharmacy, Shenzhen Technology University, Shenzhen 518118, China; jx19962021@163.com (X.J.); 2201459@stu.neu.edu.cn (J.S.); wuyijian0101@163.com (Y.W.); a2313066413@163.com (S.F.); sunzeao1010@163.com (Z.S.); hbqinghe0806@163.com (Y.H.); yumengxue12@163.com (M.Y.); 13420017630@163.com (R.H.); 2 College of Materials and Energy, Jiangxi Science and Technology Normal University, Nanchang 330013, China; 3 College of Life and Health Sciences, Northeastern University, No. 3-11, Wenhua Road, Shenyang 110819, China

**Keywords:** *Aspergillus oryzae*, secondary metabolism, secondary metabolites, regulatory gene

## Abstract

The filamentous fungus *Aspergillus oryzae* (*A. oryzae*) has been extensively used for the biosynthesis of numerous secondary metabolites with significant applications in agriculture and food and medical industries, among others. However, the identification and functional prediction of metabolites through genome mining in *A. oryzae* are hindered by the complex regulatory mechanisms of secondary metabolite biosynthesis and the inactivity of most of the biosynthetic gene clusters involved. The global regulatory factors, pathway-specific regulatory factors, epigenetics, and environmental signals significantly impact the production of secondary metabolites, indicating that appropriate gene-level modulations are expected to promote the biosynthesis of secondary metabolites in *A. oryzae*. This review mainly focuses on illuminating the molecular regulatory mechanisms for the activation of potentially unexpressed pathways, possibly revealing the effects of transcriptional, epigenetic, and environmental signal regulation. By gaining a comprehensive understanding of the regulatory mechanisms of secondary metabolite biosynthesis, strategies can be developed to enhance the production and utilization of these metabolites, and potential functions can be fully exploited.

## 1. Introduction

*Aspergillus oryzae* is a common aerobic fungus that has been used in the food industry for over a thousand years. Notably, it is the oldest and most widely used microorganism in the brewing industry. In addition, the US Food and Drug Administration has classified *A. oryzae* as “generally recognized as safe” (GRAS), and the World Health Organization has acknowledged its safety [1,2,3]. The safety of phospholipase A1, a food enzyme produced by the transgenic strain NZYM-LJ/NZYM-PP of *A. oryzae*, has also been validated [4,5]. Though widely distributed and rapidly multiplying, *A. oryzae* exhibits slight variations in colony growth among different strains. In China and Japan, *A. oryzae* plays a crucial role in traditional brewing technology and is even regarded as a “national fungus” in Japan [6].

The *A. oryzae* RIB40 genome, which measures 37.9 Mb, was first sequenced in 2005 [7]. Compared to other species (Table 1), *A. oryzae* has a bigger genome, primarily due to the amplification of metabolic genes, including those related to secretory hydrolases, transporters, and primary and secondary metabolism [7,8]. Not only does *A. oryzae* possess a remarkable protein secretion function, but it also exhibits a closer resemblance to natural eukaryotic genetic products [9,10]. Moreover, with its extensive history of industrial application and capacity for large-scale fermentation, it represents an ideal and efficient system for the expression of foreign genes [11,12,13]. Useful proteins have been successfully generated from animal and plant sources using *A. oryzae* [14]. Additionally, the successful heterologous expressions of important structural natural products documented in the literature have been achieved through *A. oryzae* (Table 2). Furthermore, the heterologous expressions of these compounds have resulted in higher yields than those produced using their original strains [15]. For example, the heterogeneous expression of the trili biosynthetic gene clusters of *Trichoderma reesei* in *A. oryzae* produced new compounds (two acyl tetramic acids), indicating the fungal host diversity in catalytic reactions [16].

Superbugs constantly emerge and proliferate due to the misuse of antibiotics, inflicting severe damage to human life and health. The filamentous fungi genomes consist of numerous gene clusters responsible for the biosynthesis of secondary metabolites compared to Actinobacteria, indicating their higher potential for producing active metabolites. Notably, secondary metabolites facilitate biological signal transduction throughout an organism’s life cycle and produce “defense compounds” that enable adaptation to environmental changes [17,18,19]. Various microorganisms synthesize diverse secondary metabolites intracellularly or extracellularly during specific temporal windows, which function in signal transduction pathways [20]. In Aspergillus, the biosynthesis pathway of secondary metabolites is regulated by pathway-specific transcription factors and global regulatory factors such as environmental conditions and epigenetic modifications [21,22]. The secondary metabolites of *A. oryzae* contain numerous physiologically and pharmacologically active compounds and a few mycotoxins [23,24,25]. These products play an integral role in the evolution of *A. oryzae* and its interactions with other organisms and the environment.

**Figure 1:**
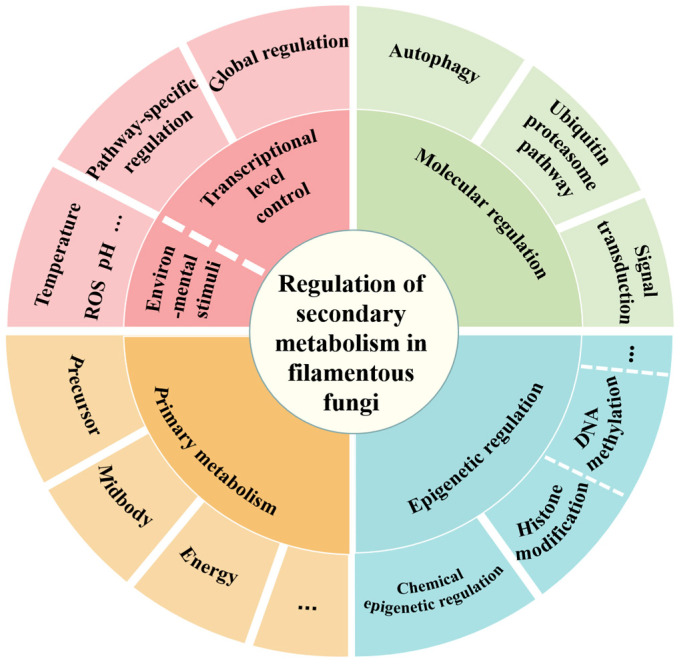
Regulatory network of secondary metabolism in filamentous fungi.

Deciphering the *A. oryzae* genome, especially with the identification of the many gene clusters responsible for the synthesis of secondary metabolites, has provided a genetic basis for the abundant biosynthesis of secondary metabolites through this microorganism. For example, kojic acid, one of the primary secondary metabolites produced by *A. oryzae* [26], is widely used in cosmetics as the main component of various whitening products [27]. Kojic acid and its derivatives also serve as a raw material for antibacterial, antifungal, and anti-inflammatory agents in the pharmaceutical industry [28,29,30]. Researchers have also discovered polysaccharides with anti-tumor activity [31,32], active compounds against Alzheimer’s disease, antibacterial compounds [33,34], and enzyme inhibitors [35] from various *A. oryzae* strains. Although ergosterol, squalene, ceramide, and other common secondary metabolites are synthesized by *A. oryzae*, there is still limited knowledge on the potential of *A. oryzae* in producing other secondary metabolites, with reports that the known compounds represent only a minor fraction of the secondary metabolites produced by *A. oryzae* [3,36,37,38]. Therefore, the identification, validation, and development of more reactive molecules from *A. oryzae* are still ongoing, highlighting the vast potential of *A. oryzae* in various fields, such as medicine and agriculture. In this review, we analyzed the secondary metabolism regulation in *A. oryzae*, with a focus on three key aspects: transcriptional regulation, epigenetic modification, and stimulation through environmental signals. These three regulatory modes converge to form complex regulatory networks (Figure 1) and have been shown to affect the synthesis of secondary metabolites. This review delved into the production of secondary metabolites in *A. oryzae* at the regulatory level and proposes feasible strategies for mining potentially valuable compounds.

**Table 1:** Comparison of genome characteristics between *A. oryzae* and other related fungi.

Species	Chromosome Number	Genome Size (Mb)	GC Content (%)	Gene Annotation	Reference
*Aspergillus oryzae*	8	37.9	48.2	13120	[7]
*Aspergillus nidulans*	8	31	50.4	10560	[8]
*Aspergillus fumigatus*	8	29.4	49.9	9926	[39]
*Saccharomyces cerevisiae*	16	12.1	38.3	5885	[40]

**Table 2:** Structural types of natural products heterogeneously expressed by *A. oryzae*.

Type of Compound	Name and Structure	Expression Host	Reference
Polyketide	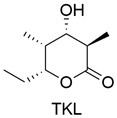	*A. oryzae* NSAR1	[41]
Peptide Compound	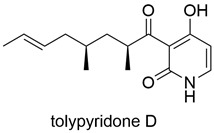	*A. oryzae* NSAR1	[42]
Terpenoid	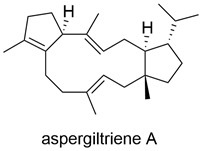	*A. oryzae* NSAR1	[43]
Other	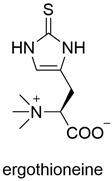	*A. oryzae* NSAR1	[44]

## 2. Transcriptional Regulation of Secondary Metabolism in *A. oryzae*

Secondary metabolism in filamentous fungi is a complex and multi-level regulatory process involving numerous enzymatic reactions. Specifically, the biosynthesis of secondary metabolites is regulated by pathway-specific transcription factors and global regulatory factors [21].

### 2.1. Global Regulatory Factors

**Figure 2:**
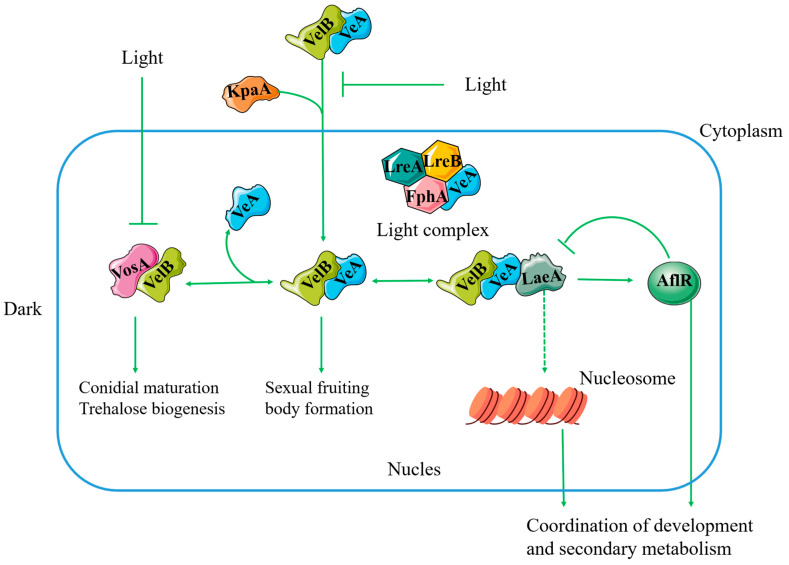
A schematic representation of the global regulation of the LaeA and Velvet family proteins. Importin alpha KapA facilitates the nuclear entry of the VelB-VeA dimer. VelB can assemble into two distinct complexes within the nucleus. The VosA-VelB dimer suppresses asexual spore formation while regulating spore maturation and trehalose synthesis. The VelB-VeA dimer interacts with the LaeA protein to form a trimer, which modulates sexual development and secondary metabolism.

Global regulation refers to the biosynthesis and morphological differentiation regulation of multiple secondary metabolites. Although genes regulating the global transcription factors do not exist in gene clusters, they regulate the transcriptional activation of various genes, thereby influencing the production of secondary metabolites. LaeA is one of the major global regulators of secondary metabolism in filamentous fungi, which was discovered in *A. nidulans* in 2004 [45,46]. It regulates the expression of multiple secondary metabolic gene clusters and influences the formation of secondary metabolites [47,48]. LaeA forms a protein complex with veA and veB in response to light and regulates the expression of the Velet family members, thus influencing secondary metabolism, growth, and reproduction (Figure 2). It also influences the morphogenesis and development of filamentous fungi by activating the expressions of silent genes to produce new metabolites [49]. For example, fungal-specific sirtuins *hstD/Aohst4* interact with laeA, with *hstD/Aohst4* acting upstream of laeA [50], altering the production of multiple secondary metabolites. Thus, sirtuins *hstD/Aohst4* are essential in the global regulation of the biosynthesis of secondary metabolites. In addition, laeA is highly expressed in *hstD/AoHst4*-deleted strains, suggesting that *hstD/AoHst4* is involved in the suppression of the *laeA* gene.

Moreover, the overexpression of *Aokap2* under the control of an *amyB* promoter of *A. oryzae* inhibits hyphal growth, conidia formation, and biomass yield with increased kojic acid production [36]. The overexpression of *Aokap2* elevates the transcription levels of *kojA*, a key gene involved in kojic acid synthesis, and laeA, a global transcriptional regulator, increasing kojic acid production. Conversely, the expression of *Aokap2* is significantly downregulated in *laeA* mutants, decreasing kojic acid production.

With the rapid development of genetic engineering techniques, many studies are adopting these techniques to select superior strains. Moreover, mutation breeding still plays an important role in obtaining high-yield strains. For example, a kojic acid-producing strain, AR-47, was obtained through the co-mutagenesis of *A. oryzae* KA-11 [51]. A transcriptional expression analysis of kojic acid biosynthesis-related genes revealed that the expressions of these genes were higher in strain AR-47 compared to those in the original strain. In addition, *kojA*, *kojR*, and *kojT* are involved in kojic acid biosynthesis. *KojR* is positioned between *kojA* and *kojT*, directly regulating *kojT* and potentially controlling *kojA* [26,52,53]. LaeA also regulates *kojA* and *kojT* by controlling the expression of kojR. Specifically, the upregulation of laeA increases the expression of *kojR*, *kojA*, and *kojT*, implying that laeA positively regulates kojic acid synthesis. As a result, the deletion of the regulatory gene *laeA* decreases kojic acid production and related gene expression, while its presence restores kojic acid production. These findings suggest that laeA expression is also regulated by other genes. However, what drives the increased expression of kojic acid biosynthesis genes remains unknown. In addition, further research is needed to establish their regulatory relationship with laeA.

The *veA* gene mediates the response to light and regulates various cellular processes, including asexual and sexual development and secondary metabolism [54]. The beta-lactam antibiotic penicillin is derived from a few filamentous fungi with specialized production ability. In *A. oryzae*, the positive regulation of the *veA* gene cluster influences gene transcription, thereby promoting penicillin synthesis [38]. Additionally, the homologous proteins laeA and velB play a role in the regulation of the *veA* gene. For example, there is a noticeable decrease in penicillin synthesis by *A. oryzae* after *veA* deletion. Notably, the expression of *ipnA* within the penicillin biosynthetic gene clusters is positively regulated by veA in *A. oryzae* and negatively regulated by veA in *A. nidulans* [38,54].

The transcription factor brlA, belonging to the C_2_H_2_ zinc finger family, plays a crucial role in conidial development and in regulating secondary metabolism [55,56]. KpeA is highly conserved in filamentous fungi and represents a novel Zn(II)_2_-Cys_6_ binding protein, exerting global regulatory control. For example, there was a six-fold increase in kojic acid production in the Δ*kpeA* strain compared to the control strain, which was accompanied by the upregulation of the *kojR* and *kojA* genes and downregulation of *brlA*, *abaA*, and *wetA* transcription levels [57]. Therefore, kpeA functions as a Zn(II)_2_-Cys_6_ binding protein in the transcriptional regulation of conidiation and the biosynthesis of kojic acid.

In summary, evidence suggests that global regulatory transcription factors play a pivotal role in the secondary metabolism of *A. oryzae*. While the modulatory mechanisms of some transcription factors remain elusive, it is clear that global transcriptional regulators can effectively control secondary metabolites in *A. oryzae*.

### 2.2. Pathway-Specific Regulators

The primary function of gene expression in the secondary metabolic pathway is to regulate the biosynthesis gene cluster through encoding genes. In fungi, the zinc finger protein family is involved in the specific regulation of secondary metabolites. For example, the C_2_H_2_ zinc finger regulatory protein encoded by *msnA* and its cognate genes are the primary transcription factors that regulate fungal cell response to external stress [58,59]. In addition, the growth diameter, spore count, and production of kojic acid are increased in *A. nidulans* and *A. oryzae* upon knocking out *msn2*. Nonetheless, the *Aomsn2* gene in *A. oryzae* regulates *kojT* expression, impacting kojic acid synthesis. The *kpeA* mutation decreases the expression of the core regulatory factor brlA and significantly increases the expression of the pathway-specific regulatory factor kojR, increasing the production rate of kojic acid compared to the control strain [57].

Aflatoxins are primarily produced by *A. flavus* and *A. parasiticus*. Belonging to the section *Flavi*, *A. oryzae* and *A. flavus* exhibit a 99.5% gene homology. However, domestication may have caused the loss of the toxin-producing ability in *A. oryzae* [60] due to the significant deletions and multiple mutations in the aflatoxin gene cluster [61]. For example, multiple mutations have occurred in the *aflR* gene, a pathway-specific regulator of aflatoxin biosynthesis. As a result, some *A. oryzae* strains do not express aflR, while others exhibit a weak expression [62]. In addition, the deletion of *ctnA*, a specific transcription factor regulating the citrinin biosynthetic pathway, significantly reduces citrinin production by *Monascus purpureus*. Conversely, the overexpression of the *ctnA* gene in *A. oryzae* leads to an approximately 400-fold increase in citrinin levels [63,64].

Pathway-specific regulators may not only regulate gene transcription within a gene cluster but also outside of it. Additionally, the diverse modes of specific regulatory factors interact to exert control. However, further research is needed to explore specific regulatory factors that affect the *A. oryzae* secondary metabolism. Understanding these control mechanisms will provide valuable insights into the study and production of secondary metabolites.

## 3. Epigenetic Regulations of Secondary Metabolism in *A. oryzae*

**Figure 3:**
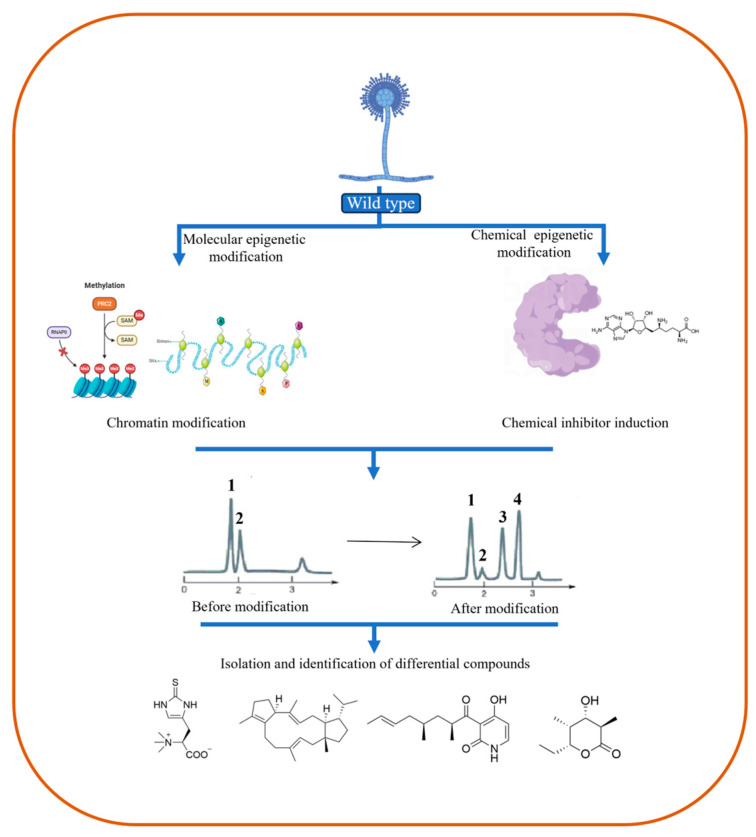
Epigenetic regulatory patterns of secondary metabolites in fungi.

The methodologies employed for the epigenetic modification of secondary metabolism regulation primarily encompass molecular and chemical epigenetic interventions (Figure 3). Molecular epigenetic regulation is based on nucleic acid modifications, including DNA methylation, histone modification, and RNA silencing systems. A significant correlation exists between secondary metabolism and epigenetic states in filamentous fungi [65]. For example, the presence of heterochromatin histone marks silences the secondary metabolism gene clusters. However, in the absence of these marks, the gene clusters are activated, altering the chromatin structure accordingly. Therefore, altering the histone modification state can regulate gene expression and induce numerous changes in metabolite spectra, ultimately activating the recessive secondary metabolite production [66,67].

### 3.1. Effect of Epigenetic-Related Genes on Secondary Metabolism

Transcription factor laeA exhibits a sequence similar to those of histone and arginine methyl transferases, suggesting that they may regulate the overall synthesis of secondary metabolites by modulating chromatin structure. In *A. oryzae*, *hstD*, a gene homologous to yeast, plays a crucial role in regulating secondary metabolism [50]. In *hstD*-deficient *A. oryzae* strains, growth is inhibited, while the yields of kojic acid and penicillin are significantly increased. However, morphogenetic defects and enhanced kojic acid production can be rescued via *hstD/Aohst4* gene insertion. Furthermore, genetic interactions between *hstD/AoHst4* and *laeA* suggest that this fungus-specific sirtuin (a member of the NAD (+)-dependent histone deacetylase (HDAC) family) coordinates fungal development and secondary metabolism by regulating laeA in filamentous fungi.

Methylation is a crucial modification that impacts gene expression. Genes homologous to *cclA*, a component of the histone 3 lysine 4 (H3K4) methyltransferase complex associated with the Set1 complex in other organisms, are present in *A. oryzae*. *A. oryzae* also contains genes homologous to *sppA* in *Saccharomyces cerevisiae*, an important component of another H3K4 methyltransferase complex [68]. The absence of both cclA and sppA hinders three methylation processes and histone H3K4, which alters the chromosome status. This influences related gene expression, ultimately leading to an improved astellolide yield. However, knowledge of epigenetic genes involved in the secondary metabolism of *A. oryzae* remains limited. Therefore, further investigations into the regulatory mechanisms governing secondary metabolism are necessary to promote research on *A. oryzae*.

### 3.2. Effect of Chemical Epigenetic Agents on Secondary Metabolism

Recent discoveries have revealed several chemical reagents that suppress the activity of enzymes involved in epigenetic modifications, thereby controlling secondary metabolism. For example, HDAC inhibitors induce the production of fungal secondary metabolites by altering histone acetylation on chromatin [69,70]. The modification transforms the silent gene locus from a hypoacetylated state to an actively hyperacetylated state, which activates gene expression and enhances the production of multiple polyketones in fungi. Therefore, modulating fungal gene expression epigenetically with HDAC inhibitors is a potentially powerful method for obtaining recessive biosynthetic natural products.

Moreover, introducing *pksCH-1* and *pksCH-2* genes from *Chaetomium indicum* into *A. oryzae* [69] revealed that *pksCH-2* was epigenetically modified to function as the silent non-reducing PKS gene encoding the common precursor 8 for the new compound, which is consistent with ChIP analysis results. However, multiple attempts to express pksCH-1 have been unsuccessful. Nonetheless, given its amino acid sequence and domain similarity to *pkeA*, it is reasonable to infer that this gene may encode a common precursor for a new compound.

Ergosterol is a crucial pharmaceutical raw material for the production of cortisol and progesterone [71,72]. Most antifungal drugs act as inhibitors of essential enzymes in the ergosterol biosynthesis pathway, which can be categorized into four distinct categories based on their specific roles [73,74,75]. For example, bioinformatic and RNA-seq analyses of the gene expression profiles in *A. oryzae* treated with tebuconazole and terbinafine revealed that there are many differentially expressed genes associated with ergosterol biosynthesis [76]. In addition, the ergosterol biosynthesis is blocked when tebuconazole inhibits *ERG11*. In contrast, terbinafine inhibits *ERG1*, decreasing ergosterol production and squalene accumulation in the plasma membrane, which increases the brittleness of the plasma membrane, impairing its structure and function. These reports demonstrate that tebuconazole and terbinafine have distinct targets and mechanisms of action [76]. Among them, 17 genes (16 downregulated and 1 upregulated) were regulated by both tebuconazole and terbinafine inhibitors, indicating that these genes may be directly targeted by ergosterol.

In summary, epigenetic regulation is a highly convenient and effective strategy for discovering new compounds. Therefore, the use of inhibitors targeting fungal gene expression for epigenetic regulation could be an effective approach to unlocking the recessive biosynthesis of natural products in *A. oryzae*. Given the limited epigenetics research in *A. oryzae*, continuous exploration in this field will establish a solid foundation for future research on Aspergillus.

## 4. Environmental Factor Regulation of Secondary Metabolism in *A. oryzae*

**Figure 4:**
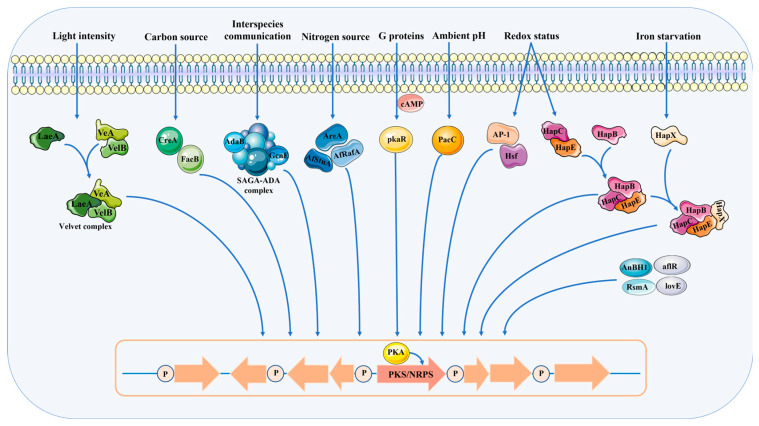
Environmental regulation of biosynthetic gene clusters for secondary metabolites in fungi. Environmental signals regulate the expressions of genes in biosynthetic gene clusters, thereby controlling the activities of key enzymes or gene expression levels in secondary metabolic pathways and ultimately affecting the synthesis of secondary metabolites. The intracellular molecule cAMP mediates the internal regulation of biosynthetic gene clusters by modulating camp-dependent protein kinase (PKA).

Fungal growth, development, and metabolism are highly susceptible to variations in environmental conditions such as medium composition, reactive oxygen species (ROS), temperature, pH level, light intensity, metal ion concentration, and interspecies interactions (Figure 4) [77,78,79,80]. Changes in these environmental factors alter the enzyme activity, subsequently affecting the diversity of secondary metabolites associated with them. In addition, the transcriptional and epigenetic regulation of genes involved in the biosynthetic pathways of *A. oryzae* is a response to environmental stimuli, with changes in the secondary metabolites being mediated by intracellular transcription factors or signal transduction pathways. Thus, environmental stimuli are essential for the production of secondary metabolites, like functional proteins, lipids, organic acids, and other secondary metabolites used in the food processing industry [3,81,82].

### 4.1. The Impact of Carbon Sources

Carbon sources play a pivotal role in fungal growth, where they serve as both carbon skeletons and energy providers. Microbial sources of carbon are classified into two: carbohydrate compounds and organic acids, along with their respective salts. These carbon sources also regulate secondary metabolite synthesis.

In shake-jar cultures of *A. oryzae* M3B9 supplemented with seven carbon sources (glucose, fructose, sorbitol, sucrose, maltose, lactose, and starch), high concentrations of kojic acid accumulated in the medium with six carbon sources except lactose, significantly inhibiting kojic acid production [83]. This may be attributed to a change in metabolic regulation. Furthermore, *A. oryzae* M3B9 efficiently utilized fructose to generate elevated levels of kojic acid, contradicting a previous report, which suggested that furanose-derived fructose cannot generate high concentrations of kojic acid [84].

In another study in which glucose was employed as a carbon source, *A. oryzae* produced 58.2 g/L of malic acid and 4.2 g/L of fumaric acid. However, xylose and glycerol resulted in decreased malic acid production [85]. Furthermore, glycerol had a positive impact on fumaric acid production, while xylose exhibited no significant effect. A comparative analysis of the active growth and lipid accumulation stages of *A. oryzae* BCC7051 also revealed that this strain exhibited greater efficiency in utilizing carbon sources to produce biomass and lipids in the C5 (xylose) medium compared to a C6 (glucose) medium [86]. Furthermore, when a mixture of glucose and xylose was used as the carbon source in the medium, an increased glucose ratio correspondingly increased the kojic acid yield from *A. oryzae* BCC7051. Therefore, careful selection of appropriate carbon sources can effectively enhance the production of secondary metabolites.

### 4.2. The Impact of Nitrogen Sources

Fungi require nitrogen for their growth and reproduction. Nitrogen also regulates the fungal secondary metabolism. Although filamentous fungi are capable of absorbing both ammonium nitrogen (NH_4_^+^) and nitrate nitrogen (NO_3_^−^), NH_4_^+^ is the preferred inorganic nitrogen source for cell growth [87,88]. Aspergillus utilizes various nitrogen-containing compounds as sole nitrogen sources, including ammonia, nitrate, and nitrite. Aspergillus growth is regulated by global transcription factors involved in nitrogen metabolism. The *areA* gene encodes a nitrogen regulatory protein that activates the transcription of numerous structural genes encoding enzymes that catabolize nitrogen sources under limited nitrogen conditions, thus promoting the availability of favorable nitrogen sources and suppressing the expression of enzymes required for non-favorable nitrogen source catabolism [89,90].

β-lactam biosynthesis is a non-ribosomal peptide synthase system responsible for the production of β-lactam antibiotics, with ACV synthase as the key enzyme in its biosynthetic pathway. The nitrogen source plays a crucial role in determining the ACV yield in *A. oryzae* after the replacement of the native promoter with *AoPgpdA* [91]. Among the various nitrogen sources, including yeast extract, mixed nitrogen source, urea, and NaNO_3_, urea is the most suitable for both cell growth and ACV production. In addition, the amount of chitin varies with changes in nutritional supplements and environmental stress, impacting the glucosamine (GlcN) yield. GlcN is an amino monosaccharide and structural component of chitin and chitosan, with diverse therapeutic effects such as antioxidation, anti-aging, and anti-inflammation [92]. Increasing the nitrogen sources significantly increases the GlcN concentration in *A. oryzae* NCH-42, with yeast extract being the optimal nitrogen source [93]. Yeast extract and NaNO_3_ are also the optimal nitrogen sources for the production of anhydromevalonolactone (AMVL), a naturally occurring compound that can be heterogeneously expressed by *A. oryzae* MTG4 [94]. Increasing the concentration of either yeast extract or NaNO_3_ enhances AMVL yield. However, excess nitrogen sources may lead to decreased yields. Nonetheless, adding a small amount of NaNO_3_ to the standard medium results in a complete loss of kojic acid formation, indicating its inhibitory effect on kojic acid formation [52,53]. A similar outcome was observed in the *A. oryzae* RIB40 strain [95], suggesting that NaNO_3_ is not the optimal nitrogen source for the production of all secondary metabolites.

These reports suggest that nitrogen sources significantly impact *A. oryzae* growth, development, and metabolism. Therefore, the selection of the optimal nitrogen source will inevitably influence the yield of secondary metabolites. Moreover, the different nitrogen sources exert varying degrees of influence on the morphology of *A. oryzae*, impacting subsequent secondary metabolite yields [83].

### 4.3. The Impact of Temperature

Temperature significantly impacts the growth and metabolism of *A. oryzae*, with both low and high temperatures hindering mycelial growth and conidia formation [96]. Different culture temperatures primarily impact the activity of the enzymatic system in *A. oryzae*, subsequently regulating strain growth and metabolism at a molecular level to promote or hinder mold development [97,98]. For example, high temperatures denature and inactivate specific enzymes, decreasing the chemical reaction, metabolism rate, and metabolic products. Conversely, low temperatures hinder the activity of some *A. oryzae* enzymes, slowing the growth of the fungi. With a temperature growth range of 20–40 °C [99], *A. oryzae* exhibits optimal growth at 30–35 °C [100]. In addition, fermentation temperatures are typically maintained at 15–45 °C to enhance the relative concentration of flavor compounds [96].

Temperature stress primarily affects glucose, glycerolipid, and linoleic acid metabolism. Low-temperature stress upregulates trehalose synthesis and starch metabolism encoding genes [96]. Conversely, high-temperature stress suppresses the expression of genes regulating fructose, galactose, and glucose metabolism and hinders the normal functioning of the triacylglycerol pathway to decrease the triacylglycerol products [96].

Suppressing *AoAur1* gene expression decreases inositol phosphate ceramide (IPC), a signaling molecule that facilitates the adaptation of *A. oryzae* to diverse environments, but it increases the dihydroceramide and galactoceramide contents [101]. Additionally, inhibiting *AoAur1* expression upregulates genes associated with mycelial fusion, enhancing the transduction of stress signals and augmenting cellular adaptability to temperature stress. Therefore, downregulating the *AoAur1* gene and reducing IPC accumulation are the underlying mechanisms employed by *A. oryzae* to adapt to temperature stress.

Mevalonate diphosphate decarboxylase, also known as Erg19, is a crucial enzyme in the mevalonate pathway [102,103]. The *AoErg19*-overexpressed and RNAi *A. oryzae* strains exhibit reduced ergosterol content and increased sensitivity to abiotic stress [104]. Moreover, the transcription levels of *AoErg19* are decreased with an increasing salt concentration, ethanol concentration, and temperature [105,106]. Therefore, manipulating the temperature and other environmental factors modulates the transcriptional expression of *AoErg19* to regulate the biosynthesis of corresponding secondary metabolites. The yield of *A. oryzae* NCH-42 was low at 20 °C, while the yields of GlcN were high at 25 °C and 30 °C [93]. Additionally, a study using *A. oryzae* to produce fructo-oligosaccharides revealed that the enhanced activity of mutant V242E is not affected by reaction temperature or other environmental factors [107]. Therefore, not all secondary metabolites are sensitive to temperature, and there are different mechanisms in response to temperature stress.

Overall, there are variations in the impact of temperature stress on the secondary metabolites of *A. oryzae*, with both positive and negative regulation being observed in metabolite production. Therefore, metabolic gene expression in *A. oryzae* can be influenced to enhance output by regulating temperature changes to meet the different requirements for various secondary metabolites.

### 4.4. The Impact of Other Factors

Other factors influencing metabolite production include pH, metal ions, oxidative stress, fermentation time, and so on. Most of these factors exert their influence on the secondary metabolism of *A. oryzae* by regulating transcription factors and the metabolic gene expression. The effect of pH is mediated by *PacC*, one of seven genes involved in pH regulation [108,109]. In an acidic environment, *PacC* remains inactive; however, in an alkaline environment, PacC acts as an activator of alkaline genes and a repressor of acidic genes. Generally, the *pal/PacC* pathway regulates the synthesis of extracellular hydrolases, including proteases, based on the environmental pH [110]. The pH also influences GlcN yield. At pH 2.5, *A. oryzae* NCH-42 produces the highest GlcN yield, with GlcN concentration and content being 4.1 and 2.4 times higher than those at pH 4~7, respectively [93]. However, excessive acidity (pH 2.0) significantly reduces the GlcN yield of *A. oryzae* NCH-42, making it inefficient under acidic conditions.

Among the metal ions influencing metabolite production, Mg^2+^ is essential for PPTase activity. In the absence of Mg^2+^, the biomass titer of ACV obtained under neutral pH conditions is significantly reduced. However, when the concentration of Mg^2+^ is increased to 10 mM, it exerts a strong positive regulatory effect on ACV production [91].

Moreover, oxidative stress responses serve as protective mechanisms against ROS, which induce cellular damage and dyshomeostasis. YAP1 and SKN7 transcription factors are responsible for the expression of crucial genes that encode enzymes essential for ROS detoxification. For example, in *A. oryzae*, the expression of redox-related genes and YAP1 and SKN7 transcription factors are upregulated under hydrogen peroxide and menadione sodium bisulfite stimulation, resulting in increased glutathione content [111].

The high content of aldehydes significantly contributes to the overall volatile flavor of *A. oryzae* 100-8. Furthermore, *A. oryzae* 100-8 produces a notably higher proportion of aldehydes compared to *A. oryzae* 3.042. Compared with *A. oryzae* 3.042, *A. oryzae* 100-8 exhibits faster growth and produces elevated concentrations of aldehydes, esters, and furans [112,113].

Overall, the secondary metabolites of *A. oryzae* are influenced by environmental factors other than carbon and nitrogen sources and temperature. For example, there are significant variations in 76 secondary metabolites, including volatile components of branched lipids and benzene series produced by *A. oryzae* exposed to different temperatures and pH levels for varying durations. The high-temperature conditions induced a notable reduction in the composition of linear volatile lipids, while low pH substantially increased the furan compound production [114]. In conclusion, diverse secondary metabolic pathways exhibit distinct responses to various environmental factors. Therefore, modifying environmental conditions is a feasible and effective approach for enhancing the secondary metabolism of *A. oryzae*.

## 5. Conclusions and Perspective

In this review, we described how the regulation of biosynthetic gene clusters affects secondary metabolite synthesis in *A. oryzae*, with a focus on transcriptional regulation, epigenetic regulation, and environmental signal regulation. Compared to primary metabolites, secondary metabolites are greatly diverse and play a more significant role in response to changes in biotic and abiotic factors. By regulating the metabolic pathways and intracellular material transformations, energy transfers can be directed toward the desired pathway, ultimately increasing the secondary metabolite output. The regulation of functional gene expression lies at the heart of *A. oryzae* secondary metabolism, which is influenced by changes in extracellular environmental factors and intracellular genes. These changes are integrated into a multi-level regulatory network through modifications in chromatin structure, signal transduction pathways, and the activity of transcription factors. Improving the production of secondary metabolites is an important aspect of biological and pharmaceutical research, and mining these compounds can expand their potential applications in various fields.

**Figure 5:**
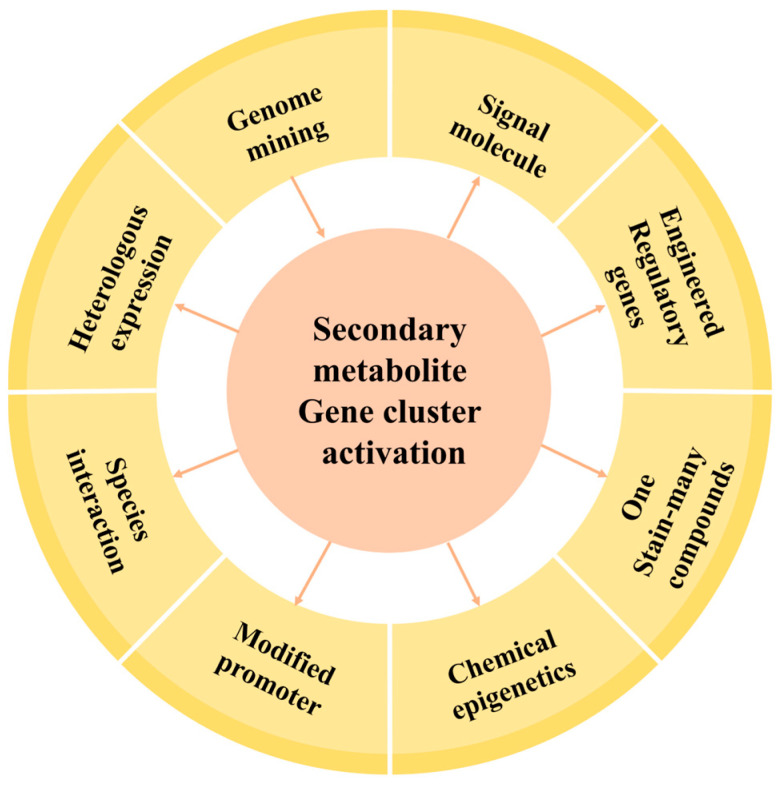
Activation of the recessive gene cluster and strategies for mining novel compounds in *A. oryzae*.

*A. oryzae* can activate the expression of silent gene clusters and produce new metabolites through heterologous expression, directional modification, and interspecific interaction (Figure 5). Progressive and deeper research on the secondary metabolic regulatory network of *A. oryzae* can effectively guide us toward a more rational application of its secondary metabolites, thereby expanding the *A. oryzae* industrial scope. Katayama et al. developed a more efficient genetic engineering technique for *A. oryzae* based on the CRISPR/Cas9 system and the recycling of autonomously replicating plasmids utilizing AMA1 [115]. This advancement increases the mutagenesis efficiency from 10–20% to 50–100% [115,116]. Utilizing this genetic engineering approach in *A. oryzae* enables efficient marker-free multi-gene deletion/integration, suitable for molecular breeding aimed at a high-level heterologous production of proteins and secondary metabolites in filamentous fungi [115]. In fully utilizing the CRISPR-Cas system, *A. oryzae* genome editing can be further enhanced, thus laying the foundation for its application in the increasingly advancing gene editing technology and high-throughput screening technology. Metabolite enhancement should not only focus on metabolic pathways, but also consider the organellar distribution of metabolites and biological reactions. This can be achieved through genome-wide analysis, metabolic network modeling, and metabolomic analysis. In addition, combined with the high-throughput omics data, the application of machine learning and artificial intelligence in the design and remodeling of metabolic pathways of *A. oryzae* can be explored, so as to formulate metabolic engineering strategies to improve the metabolic capacity and efficiency. Furthermore, whether group-sensitive effects and specific molecular pathways regulate secondary metabolism in *A. oryzae* is worth investigating. Despite the abundant availability of microbial resources, there are still numerous unexplored functions that can be discovered through ongoing research on Aspergillus. Therefore, ongoing in-depth studies on the metabolic regulation and heterologous expression in *A. oryzae*, aiming for breakthroughs in its development and application, are necessary.
